# Medical Problems of Aviation

**Published:** 1918-11

**Authors:** A. Bernard

**Affiliations:** Le Progres Medical


### ABSTRACTS

### Medical Problems of Aviation.

A. BERNARD, Le Progres Medical, May 11, 1918.

Racial aptitude plays a factor, especially in regard to habits of sportsmanship. Analogously, the cavalry is more likely to furnish candidates than the infantry and, till recently, Germany has drawn almost entirely on the former. Good aviators are always bad sailors, on account of the development of the sense of equilibrium. The best age is from 18 to 30. Weight and height are of little importance but the height should not be less than 1.55 (about 62 inches) or the aviator will have difficulty in looking over the sides of his cradle. Deformities and surgical lesions are of indirect importance, as in affording a subsequent pretext for leaving the air service, in interfering with the solidity of the abdominal wall since the displacement of the viscera in a sudden manoeuvre leads to syncope or lypothymia. Thoracic lesions are of importance mainly as they interfere with respiration as the oxygen is diminished by half at 5000-6000 meters. Anyone who cannot hold the breath at full inspiration for 45 seconds after full exhalation is unfit and most good aviators can hold the breath for a minute without discomfort. Injuries to the head are also likely to be significant, though not necessarily, by indicating cerebral change such as to cause slowness of perception, rapid fatigue, exaggerated emotional state, or sensibility to changes in atmospheric pressure. So far as the limbs are concerned, it must be remembered that almost all motions of the aviator require the action of the arms and hands above the shoulders. Amputation of the leg may not interfere with the functions of the pilot as an artificial limb can be attached to the pedal. Limitation of ankle movements leads to assignment to a hydroplane or aeroplane less rapid than a chaser. Subjects who have had infantile paralysis are barred because they are predisposed to trophic troubles excited by cold.

Excessive use of tobacco, alcohol, or other drugs bars from the service but teetotalism is not insisted on by all though none allow tobacco or alcohol immediately before flights. Syphilis and malaria, properly treated, do not bar from service. Epilepsy, tuberculosis, bronchitis, pleurisy, asthma, do. Seasickness does not bar (nausea from test movements in the Barany chair being rather considered as an index of delicacy of the sense of equilibrium) as it is rarely encountered during flights though some pilots vomit after landing. Sugar and albumin in the urine are positive disqualifications. All forms of cardiac disease and functional disturbance disqualify as do even conditions of unstable vascular equilibrium, Raynaud's disease for instance predisposing to frost bite.

Nervous instability as indicated by exaggerated knee jerk, tremors, insomnia and agitation must be carefully excluded, there being a special malady known as aeronurosis.

Vision should be perfect, without lenses, as the latter may be broken or clouded. Some candidates with defective vision have adapted themselves and become excellent pilots. Not only must vision be perfect in the ordinary military sense but it must be perfect for both eyes together and for each separately; stereoscopic vision must be good to allow for estimating distances, color sense to distinguish insignia, signals, etc., night blindness must be excluded; larval cases of hypermetropia and astigmatism may cause bad landings.

Deafness of either ear disqualifies, not so much because the aviator depends on observation of sounds but for the very simple reason which is apt to be overlooked even by those accustomed to the use of automobiles: it is absolutely necessary that both pilots and even mechanics who do not fly shall be able to hear how the engine is working. Deviation of the nasal septum tends to bad headache on landing. Large tonsils or other causes of mouth breathing are serious as predisposing to anginas, especially from the wind of helices in front. Otitis meda suppurativa, perforation of cicatrix of the drum rejects, partly because painful affections may develop from the noise of the motor or sudden changes of altitude.

Equilibration and muscular sense are highly important yet it must be admitted that the orientation depends largely on the eyes, skilled aviators often finding that in clouds, they have been flying with one wing low or have been mounting or descending without realizing it. Graeme Anderson, bandaging his eyes, undertook to describe to the pilot, by a telephone, the changes of direction and found that while at first his descriptions were correct, he soon lost the power to orient himself. The various Barany tests and use of hot and cold water in the ears are alluded to briefly. A rough substitute for the chair is to blindfold the candidate and have him spin on one foot.

Psycho-motor tests depend largely on chronometer studies of response to various reflexes, as a revolver shot, moving needle or a touch of the head or hand. Hipp's chronoscope and d'Arsonval's chronometer are mentioned. Gemelli also has a modification. The normal response to visual stimuli is 0.19 second, retardation 0.22-0.45; to auditory 0.14, retardation 0.20-0.39; to tactile 0.14, retardation 0.20-39, these retardations being abnormal but apparently actually observed at times. Emotional tests. The aviator should not show undue reflex response to stimuli in the respiration and circulation, for obvious reasons. The test is usually made by recording the reflex from a revolver shot on drums. Tachypnoea and vaso-constriction, while normal, should be of short duration. Power of observation—attention—is tested by Rossolimo's method.

The medical examination of aviators cannot be terminated by a preliminary test, however thorough but must continue during the training period. The surgeon on duty at an aviation camp must also study, the causes of every accident and try to determine methods of avoiding them, as well as develop methods for prompt medical and surgical aid. He must also study the psycho-physiologic reactions of each student, especially in regard to pulse, arterial tension, respiration and audition and equilibrium. Delicate recording apparatus is desirable. The pulse is notably accelerated up to heights of 1000 meters, slowed from heights of 1000 up to 1400 meters, then again accelerated. It is accelerated by changes of momentum. In descending, it is first accelerated, then slowed. Respiration in general follows the change of frequency of the pulse but is always accelerated by altitude on account of the diminution of oxygen in a unit volume of air. Arterial tension diminishes up to 300-500 meters, then rises slowly. It is diminished in descending. In mounting, up to 1500 meters, all the upper respiratory passages and even the ears, become congested. Relief is obtained by deep breathing and Valsalva's experiment. These troubles disappear at 4000 meters, re-appearing on descending. More or less deafness and even bleeding from the ears occurs on landing. These troubles, due to differences in atmospheric pressure, are more pronounced if the aviator has some degree of otitic sclerosis, malformation of the nasal fossae, etc. In mounting, Valsalva's test and in descending, Toynbee's, should be employed. Complete rest for 15-30 minutes after landing is very useful and a month's rest causes the disappearance of persistent troubles of this nature.

The causes of accident are very numerous. Defects of parts of the aeroplane are now rare. Stopping of the motor is no longer a cause of death if it occurs at a sufficient height so that the pilot may volplane and choose a suitable landing place but it is dangerous at small distances from the ground and especially shortly after starting. Among 58 accidents collected by Graeme Anderson, 2 occurred in the air and 46 in landing which is the bete noir of aviator students. In general accidents are due to an error in judgment or to a defect of binocular vision on the part of the pilot. Nervous fatigue, especially and quickly developed in the inexperienced, is the principal cause of "losing one's head." Thus short flights should always be enjoined at the beginning.

To provide immediate assistance in case of accident, every camp should have an observer who communicates by telephone to the surgeon on duty who has an automobile in readiness with a fully equipped surgical and medical chest. A very practical addition to this is a tool kit including cutting pincers, hammer, fire extinguisher, etc. The camp is divided into sectors with established boundaries, in order to locate accidents without delay.

